# POLR3-Related Leukodystrophy: A Qualitative Study on Parents’ Experiences With the Health Care System

**DOI:** 10.1016/j.pediatrneurol.2025.02.011

**Published:** 2025-02-27

**Authors:** Adam Le, Kelly-Ann Thibault, Pouneh Amir Yazdani, Enrico Bertini, Francesco Nicita, Daniela Pohl, Sunita Venkateswaran, Stephanie Keller, Deborah Renaud, Dolores Gonzales Moron, Marcelo Kauffman, Danilo De Assis Pereira, Adeline Vanderver, Maxime Morsa, Geneviève Bernard

**Affiliations:** a Child Health and Human Development Program, Research Institute of the McGill University Health Centre, Montréal, Québec, Canada; b Department of Neurology and Neurosurgery, McGill University, Montréal, Quebéc, Canada; c Faculté de Médecine, Université de Montréal, Montréal, Québec, Canada; d Unit of Neuromuscular and Neurodegenerative Diseases, Bambino Gesù Children’s Hospital, IRCCS, Rome, Lazio, Italy; e Division of Pediatric Neurology, Children’s Hospital of Eastern Ontario, University of Ottawa, Ottawa, Ontario, Canada; f Division of Neurology, Children’s Hospital, London Health Sciences Center, Schulich Medicine and Dentistry, Western University, London, Ontario, Canada; g Division of Pediatric Neurology, Department of Pediatrics, Children’s Healthcare of Atlanta and Emory University, Atlanta, Georgia; h Division of Child and Adolescent Neurology, Department of Neurology and Pediatrics, Mayo Clinic College of Medicine and Science, Rochester, Minnesota; i Neurogenetics Unit, Department of Neurology, Hospital JM Ramos Mejia and CONICET-Universidad Austral, Buenos Aires, Argentina; j Department of Human Reproduction and Childhood, Pontifical Catholic University of São Paulo, Sorocaba, São Paulo, Brazil; k Division of Neurology, Department of Pediatrics, Children’s Hospital of Philadelphia, Philadelphia, Pennsylvania; l Department of Neurology, Perelman School of Medicine, University of Pennsylvania, Philadelphia, Pennsylvania; m Adaptation, Resilience, and Change Research Unit, Department of Psychology, Université de Liège, Liège, Belgium; n Department of Pediatrics, McGill University, Montréal, Québec, Canada; o Department of Human Genetics, McGill University, Montréal, Québec, Canada; p Division of Medical Genetics, Department of Specialized Medicine, McGill University Health Centre, Montréal, Québec, Canada

**Keywords:** POLR3-related leukodystrophy, Thematic analysis, Health care experiences, Rare disease, Parents/caregivers, Access to care, Quality of care

## Abstract

**Background:**

POLR3-related hypomyelinating leukodystrophy (POLR3-HLD) is a rare, inherited neurodegenerative disorder affecting white matter development of the central nervous system. This disorder is characterized by hypomyelination, hypodontia, and hypogonadotropic hypogonadism (4H leukodystrophy). Patients with POLR3-HLD require complex and specialized care; however, due to its rarity and limited awareness, parents often assume additional roles as experts and advocates for their child(ren). We aimed to understand parents’ experiences navigating the health care landscape and to identify potential targets for improvement.

**Methods:**

Research team members conducted semi-structured interviews with parents of patients with POLR3-HLD. Interview questions focused on the diagnostic odyssey, availability and access to care, and the perceived quality of care. Interviews were recorded, transcribed, coded, and analyzed using reflexive thematic analysis, and themes surrounding parents’ health care experiences were developed.

**Results:**

Nineteen semi-structured interviews were conducted with an international cohort of 24 parents between March and October 2023. Four themes were developed: existing barriers in accessing care, limited knowledge in diagnosis and care, parents as experts and advocates of their child(ren)’s care, and perceived superior care by leukodystrophy specialists. Many parents expressed feeling alone and uncertain, with little guidance provided to them. They also identified perceived gaps in care and challenges faced but found comfort when treated by leukodystrophy experts in specialty clinics.

**Conclusions:**

This study will help better inform health care providers, administrators, and policymakers to expand and improve access to quality care for patients with POLR3-HLD and their families. These conclusions may also be generalizable to other rare diseases.

## Introduction

RNA polymerase III-related hypomyelinating leukodystrophy (POLR3-HLD; MIM: 607694, 614381, 616494, 619310), one of the most common hypomyelinating leukodystrophies,^1–6^ is an autosomal recessive disorder caused by biallelic pathogenic variants in genes encoding RNA polymerase III (Pol III) subunits (*POLR3A*,^4^ *POLR3B*,^5^ *POLR1C*,^7^ *POLR3K*,^8^ and *POLR3D*^9^). POLR3-HLD is also known colloquially as *4H leukodystrophy* due to its cardinal clinical features of hypomyelination, hypodontia, and hypogonadotropic hypogonadism.^3,10^ Patients with POLR3-HLD can experience an array of neurological or non-neurological characteristics.^11,12^ Neurological manifestations typically present as developmental delay, motor dysfunction (with primary cerebellar involvement), and cognitive impairment.^10,11,13,14^ Non-neurological features include dental,^11,15^ ocular,^16^ and endocrine abnormalities.^17^

Given its rare nature, a specific diagnosis is often difficult to determine.^1,18–21^ Patients can experience an extensive “diagnostic odyssey”,^22^ resulting in significant stress, uncertainty, and a heavy financial burden on families.^23^ With no approved curative treatment, POLR3-HLD disease management is largely symptomatic,^10^ requiring complex and multidisciplinary care.^24^ Parents can spend extensive time as communicators and coordinators of their child(ren)’s care and are often required to become experts and advocates due to the scarcity of health care practitioners (HCPs) knowledgeable about POLR3-HLD.^5^ Specialized leukodystrophy (LD) centers and clinics offer expert care, information, and support to patients with LDs; however, they are primarily located in the United States, which poses a major accessibility barrier for many patients globally.

This is the first study to explore patients with POLR3-HLD’s health care experiences, bridging the gap in understanding across diverse international perspectives. A qualitative research approach, utilizing in-depth interviews, is ideal to uncover the nuanced insights and personal challenges families face in navigating care pathways for this rare disorder. The overarching goal of this study is that parents’ shared experiences will highlight potential targets to improve the access and quality of care for patients with POLR3-HLD and avenues for improving the management of rare pediatric conditions.

## Methods

### Study design

We conducted in-depth, semi-structured interviews with parents of patients with POLR3-HLD. This study was approved by the Research Ethics Board (REB) of the McGill University Health Centre Research Institute (MUHC-RI) (2020–6222).

### Participants

Twenty-four parents (18 mothers and six fathers) were recruited for this study using purposive sampling.^25–27^ Participants were recruited from the MyeliNeuroGene Biobank (approved by the MUHC-RI REB, 2019–4972). Participants were eligible if they were the parent(s) of a patient with POLR3-HLD, could speak English or French, and were able to come to the Montreal Children’s Hospital or reached virtually for the interview. Purposeful sampling^25–27^ was used to ensure diversity across geographic location, socioeconomic status, ethnicity, and patients’ ages. The MyeliNeuroGene Lab social media pages were also used to promote participation and recruitment. All participants were contacted by email, informed about the research study, and gave written consent.

### Interview guide

A semi-structured interview guide was developed in English by research team members and translated into French (Supplemental Material). Interview questions focused on the diagnostic odyssey, the availability and access to care, and the perceived quality of care. It was then pilot-tested with a parent of a child with Pelizaeus-Merzbacher-like disease, a LD with a similar clinical phenotype to POLR3-HLD, and no changes were suggested.

### Researchers

Our research team was composed of a graduate student (A.L.), a medical student (K.-A.T.), a pediatric neurology resident (P.A.Y.), and an international group of clinician experts in LDs (E.B., F.N., D.P., S.V., S.K., D.R., D.G.M., M.K., D.D.A.P., A.V.), and it was supervised by an expert in qualitative health research (M.M.) and a pediatric neurologist and clinician-scientist specialized in LDs (G.B.). G.B. is the director of the Leukodystrophies and Neurometabolic Disorders Clinic at the Montreal Children’s Hospital.

### Data collection

Participants completed a sociodemographic questionnaire before the study interview. A.L. or K.-A.T. conducted semi-structured interviews with parents virtually or in person at the Montreal Children’s Hospital between March and October 2023. Interviews were completed with one parent only or both parents together, based on participants’ preferences. Interviews lasted between 30 and 120 minutes. These interviews were recorded, transcribed verbatim, reviewed, and de-identified by A.L. or K.-A.T. Interviews were completed until data saturation was reached.^25,26^

### Data analysis

We performed reflexive thematic analysis to develop themes from parents’ personal experiences and perspectives.^28–30^ De-identified interview transcripts were uploaded to NVivo 14,^31^ and coded independently by A.L. and K.-A.T. through a predominately inductive approach. Discussions were held every two to three coded interviews to present the codes, the reasoning employed, and ideas about the data. Once coding was complete, A.L. developed larger patterns across the dataset and potential themes of meaning. Given the exploratory nature of our study and the rarity of POLR3-HLD, we adhered to a fully qualitative (“Big Q”) approach, following the latest recommendations against statistical analyses in reflexive thematic analysis.^30^ This method prioritizes understanding personal meanings over post-positivist quantification, ensuring a comprehensive interpretation of the dataset, guided by a relativist ontology and constructionist epistemology.^30,32^

## Results

Nineteen semi-structured interviews with 24 parents of patients with POLR3-HLD were completed; this represents 19 families of 22 patients aged between three and 34 years. Three families had two affected children. Household income ranged from under $7000 to above $250,000 (CAD [Canadian dollar]). The participants’ regions of origin, current continent of residence, and distance from the closest LD center or clinic are represented in Table 1.

Four main themes were developed through analysis. These include (1) existing barriers in accessing care, (2) limited knowledge in diagnosis and care, (3) parents as experts and advocates of their child(ren)’s care, and (4) perceived superior care by LD specialists. Representative quotes from these themes are included in Table 2.

### Theme 1: Existing barriers in accessing care

Parents of patients with POLR3-HLD identified several barriers when seeking specialized care for their children. One major hurdle is the distance they must travel to reach LD centers/clinics, which is financially burdensome and physically and mentally exhausting. Parents also faced difficulty accessing local care, citing extensive challenges in scheduling appointments with HCPs across their cities. Long appointment wait times were noted as a key point of contention with parents, often waiting many months to be seen.

The trade-offs between public and private health care systems in parents’ regional countries were also extensively discussed. Those relying on predominately public systems valued the cost-free services but dreaded its slow-moving pace. This led them to frequently turn to private alternatives for rehabilitation services, therapies, and medical testing, which they described as costly, imposing financial strain on their families when seeking necessary care. Parents faced a considerable challenge in navigating the insurance landscape within the private health care system. Substantial time was devoted to persuading insurance providers about the essential nature of equipment and therapies. POLR3-HLD being absent from numerous approved insurance lists meant that many recommended treatments by HCPs remained uncovered, forcing parents to expense many things out of pocket.

Financial security greatly increased parents’ ability to access care for their child(ren), enabling access to private health services, the ability to afford necessary equipment, cover travel expenses to be seen at LD centers/clinics, and limit their working hours to serve as caretakers. The implementation of telemedicine also helped tremendously, providing parents with greater flexibility in managing their child(ren)s care through virtual appointments, and has increased accessibility to distant LD specialists.

### Theme 2: Limited knowledge in diagnosis and care

Parents expressed many frustrations with the health care services they have received over the years, attributing much of their dissatisfaction to HCPs’ perceived limited knowledge and understanding of POLR3-HLD. Many experienced an extensive diagnostic odyssey from months to years as HCPs struggled to identify the condition, resulting in negative test results and incorrect diagnoses. With this limited understanding, few parents received comprehensive information following the diagnosis or guidance on what to expect. Genetic counseling and supportive interventions like social work and psychotherapy were also notably absent, leaving parents to grapple with the overwhelming news on their own.

Parents reported dissatisfaction regarding the perceived limited recommendations from HCPs. There is a perceived lack of awareness among HCPs regarding the progression of their child(ren)’s condition and the optimal approaches to address emerging challenges. HCPs sometimes failed to recommend appropriate therapies, leaving parents to seek information independently. This lack of guidance contributed to the frustration expressed by parents who desire a more comprehensive and informed approach to their child(ren)’s health care. Moreover, parents emphasized the rarity of referrals to LD centers/clinics, highlighting a perceived deficiency in HCPs’ awareness of specialized resources and expertise.

This limited understanding is exacerbated by a frequently perceived lack of interest by HCPs to learn about POLR3-HLD and understand parents’ concerns. This lack of interest often resulted in parents feeling as though HCPs dismissed their input and treated them as overbearing or paranoid. Parents felt that HCPs failed to recognize the depth of understanding they had developed about their child(ren)’s health. This recurrent pattern left them feeling marginalized and added to the challenges they faced in advocating for their child(ren)’s comprehensive care needs.

### Theme 3: Parents as experts and advocates of their child(ren)’s care

With little support provided to them due to the perceived limited expertise of many HCPs about POLR3-HLD, parents often felt required to become experts on their child(ren)’s condition and advocate heavily for them. Parents cited having to quickly learn to describe their child(ren)’s condition so that HCPs might hear their concerns. They also often had to request certain treatments or therapies as their HCPs were not recommending them. From the start, parents felt they needed to become informed and take initiative. Unfortunately, they underscored the limited support provided for parents to understand the diagnosis or navigate the health care system, leaving them to figure everything out on their own.

Parents described dedicating extensive time and effort to manage their child(ren)’s health care, and a recurring source of frustration stems from the perceived insufficient communication among HCPs on their child(ren)’s health care teams. Parents frequently found themselves acting as intermediaries, conveying messages between HCPs regarding discussions and recommendations, and providing updated medical records. This communication gap also extended between local health care teams and LD centers/clinics, where critical information often remained unshared.

Additionally, parental background in health care settings appeared to be an important asset in understanding and coping with the diagnosis and played a significant role in helping them access care and navigate the health care system.

### Theme 4: Perceived superior care by leukodystrophy specialists

The perceived superiority of care received by LD experts at LD centers/clinics was highly acknowledged by parents. Parents observed noticeable improvements in care from diagnosis and beyond, much of which they attributed to the greater understanding and experience of these HCPs. Parents recall significantly different experiences when receiving a diagnosis at an LD center/clinic. In addition to a markedly shorter diagnostic period, parents felt almost reassured when receiving the news from these specialists, who supported them in coming to a clear understanding of their child(ren)’s condition and provided the next steps to follow.

Parents shared a sense of security when seen at LD centers/clinics, describing these specialized facilities as the best possible place for their child(ren). The LD specialists offered unparalleled expertise and care, distinguishing themselves by providing insights and recommendations that parents often took back to their local HCPs. Many parents appreciated the comprehensive care received from these specialists, which often extended to the well-being of parents themselves. These appointments helped parents feel equipped with a proactive approach to their child(ren)’s care. Importantly, this perceived benefit was not exclusive to large-scale multidisciplinary LD centers. Parents seen by LD specialists at independent clinics shared similar sentiments of care satisfaction, not only by the specialists themselves but also with the HCPs to which they were referred during visits.

## Discussion

This study highlights the common barriers parents of patients with POLR3-HLD face in accessing care for their child(ren) and downfalls in the perceived quality of care received. Our findings align well with previous research completed with parents of children with rare diseases (RDs)^23,24,33–39^ and contribute new insights based on the rich and unique perspective of parents of patients with POLR3-HLD.

Parents encountered difficulty when seeking specialized care for their child(ren) due to geographic limitations. With a limited number of specialized centers, many families must travel great distances; this extends diagnostic odysseys, limits parents’ and HCPs’ awareness of specialized resources, and negatively impacts the disease course for many patients.^33^ Recent work by Willmen et al.^34^ highlighted this diagnostic delay. Patients with RDs waited approximately 1546 days before being referred to an expert and only 55 days on average to receive a diagnosis by the said expert.^34^ Furthermore, in another study, patients seen at LD centers/clinics were 1.73 times more likely to be diagnosed with LD,^40^ highlighting the importance of accessible, specialized care for patients with RDs. Parents also presented specific challenges stemming from the public and private health care systems in which they found themselves. Much emphasis was placed on the difficulty in navigating the insurance landscape. Parents of children with RDs spend extensive time communicating with and convincing insurance providers of their child(ren)’s needs, often to no avail.^35^ Many RDs face the scrutiny of narrow diagnostic criteria and are absent from approved insurance lists, excluding these patients from necessary service coverage.^36^ Together, these challenges imposed financial strains on parents seeking necessary care for their child(ren). On average, patients with RDs spend three- to fivefold more on direct medical costs compared with age-matched controls,^41^ which is compounded by diagnostic uncertainty^22,42^ and the often complicated and inadequate insurance coverage.^35,42,43^ Furthermore, previous work has demonstrated that socially privileged families traveled farther to access specialized LD care compared with those on public or government insurance.^44^ This finding highlights a potential divergence in experiences among participants in our cohort. Families with the financial and logistical capacity to seek specialized care versus those without underscore the importance of addressing systemic inequities and providing additional support for underserved populations to ensure equitable access to these specialized resources.

The perceived limited knowledge about POLR3-HLD on behalf of HCPs left parents feeling alone, with little guidance provided to them throughout the diagnostic odyssey and beyond. Unfortunately, this is a common occurrence for RD families.^24,45^ Parents attributed this and much of their dissatisfaction with health care services to a perceived lack of knowledge on the part of HCPs. Too often, parents were met with little understanding or none at all, citing many encounters where HCPs had never even heard of POLR3-HLD. A study in Poland exploring RD knowledge among future HCPs demonstrated that over 95% reported insufficient or very poor knowledge about RDs and less than 7% felt prepared to provide care for these patients.^37^ Our parents stressed the lack of adequate guidance and appropriate recommendations from their child(ren)’s HCPs. In the absence of clear directives, families were left confused and alone, and this can cause distrust in the health care community.

Therefore, parents had to quickly learn about POLR3-HLD and how to advocate for their child(ren) so their concerns might be heard. Owing to limited knowledge and awareness, they were often forced to take action and initiative over their child(ren)’s health care needs. Parents of children with RDs spend extensive time learning about their child(ren)’s condition and increasing their health literacy to become experts.^38^ We see a reversal of roles, with parents often informing physicians and requesting therapies based on their informed research. This is a unique experience for parents of children with RDs and HCPs to navigate, and unfortunately, parents cite many instances in which HCPs dismiss their knowledge, leading them to feel frustrated and isolated.^24,38^

Altogether, parents acknowledged the care provided by LD experts at specialty centers/clinics for its unparalleled expertise and comprehensive approach. Parents reported shorter diagnostic periods when seen early by an expert compared with others who spent years without answers. This is consistent with findings from a cohort of patients with rare inflammatory systemic diseases, whereby a diagnosis was only reached for 21% of patients without the help of an expert and required an average of 25 appointments (and up to 109) compared with seven when seen by an expert.^34^ Parents also recalled a notably different manner in which the diagnosis was delivered; described as informative, parents felt supported and the next steps were provided for them. Specialized LD centers/clinics offer important support for patients with RDs and families alike. The multidisciplinary teams possess specific expertise necessary for proper diagnosis and effective management, as well as a deep understanding of the unique challenges faced by these patients and their families. Comprehensive social support is available and extends to the well-being of parents alike. This appreciation for LD specialists may also reflect parents’ evolving perspectives and emotional states. Some families only encountered these specialists well into their diagnostic journeys. As such, they may have felt more at ease and better equipped when seen by these specialists, having gained more knowledge and clarity about their child(ren)’s condition. This emotional evolution may complement the high-quality care provided by specialists, contributing to their overall positive experiences. Interestingly, patients seen at independent LD clinics within a hospital system described a similar care experience to those seen at large-scale LD centers. As conduits of specialized knowledge, these independent specialists may empower their non-expert colleagues to provide more effective care to patients with RDs.^46^ RD specialists often serve as key resources within hospital systems, offering guidance and acting as internal consultants for their non-expert colleagues. This knowledge transfer occurs through various means, including detailed correspondence explaining diagnostic reasoning, clinical conclusions, and disease-specific insights; the provision of relevant literature to support continued learning; and through educational initiatives, such as workshops, case discussions, and grand rounds. Most importantly, RD specialists provide management recommendations that colleagues can apply not only to individual cases but also to future patients with similar presentations. Additionally, frequent referrals for secondary and tertiary manifestations (e.g., endocrine, ophthalmologic, rehabilitation, etc.) allow these specialists to develop proficiency in managing these rare conditions over time. As a result, parents expressed satisfaction with the care received by both RD specialist and non-specialist providers, feeling heard, understood, and supported.

These findings suggest a need for improved RD knowledge among HCPs to better serve patients. RDs are currently absent (or in minimal capacity) in undergraduate and medical-level curricula, and so many HCPs are often required to learn about these on their first encounter (e.g., seeing a patient) or through voluntary training, conferences, and symposia.^47,48^ Unfortunately, this leads to ill-equipped professionals without the adequate tools to recognize and treat patients with RDs. A more robust RD education is therefore required to better inform and train HCPs, emphasizing the importance of listening to parental concerns amid unclear diagnoses. Furthermore, this may encourage students to become RD researchers and clinical specialists, leading to advancements in research and improved patient outcomes. Although large-scale multidisciplinary LD centers provide comprehensive services, their establishment often requires substantial logistical and financial resources, making them unattainable for many health care systems and inaccessible to some families due to geographic or socioeconomic barriers. The positive care experiences reported by families seen at independent LD clinics within hospital systems highlight the potential of alternative care models. These clinics, when integrated into local health care networks, could serve as an accessible and scalable solution to improve RD care by leveraging existing infrastructure and empowering non-expert colleagues. Establishing a balance between centralized centers and decentralized specialty clinics may reduce disparities in care and provide a more inclusive approach. Future studies, however, are needed to delineate the effectiveness and feasibility of these approaches.

Our study has some limitations. Inclusion criteria limited participation to English and French-speaking participants, which may have excluded native-speaking international parents with unique perspectives. Additionally, the primary sample was selected from MyeliNeuroGene Biobank participants. Previous involvement in our research program may indicate that these parents are better situated (i.e., connected to LD networks and resources), and so it would have been interesting to discuss with more families newly navigating this experience. Some parents had difficulty accurately recalling detailed events (i.e., due to time since the diagnostic odyssey). This difficulty is expected as emotional events can affect memory recall; however, the level of recall is also representative of their experience, which was accounted for throughout the analysis. Although the interview guide was collectively developed by the research team and pilot tested to limit leading questions and ensure clarity, inadvertent bias, as observed in qualitative research, may have shaped how participants framed their responses.

## Conclusion

Building upon existing literature, our study contributes new insights into the experiences of parents of patients with POLR3-HLD, shedding light on the specific challenges faced by this population. This may act as a framework for the much-needed improvements in health care delivery for patients with POLR3-HLD, and these lessons learned, if applied, may serve patients and families affected by all LDs and more broadly, RDs.

## Supplementary Material

Supplemental Data

Supplementary Data

Supplementary data related to this article can be found at https://doi.org/10.1016/j.pediatrneurol.2025.02.011.

## Figures and Tables

**Table 1.** Summary of Parent and Patient Characteristics

Parent Characteristics	Total (*n* = 24)	%

Parent sex		
Female (mother)	18	75
Male (father)	6	25
Preferred language (*n* = 19)		
English	17	89
French	2	11
Continent of residence (*n* = 19)		
North America	12	63
Europe	4	21
South America	2	11
Africa	1	5
Distance from nearest LD center or clinic (km) (*n* = 19)		
Less than 200	3	16
Between 200 and 499	8	42
Between 500 and 749	1	5
Between 750 and 1000	1	5
Above 1000	6	32
Household income (CAD) (*n* = 19)		
Less than 50,000	4	21
Between 50,000 and 99,000	4	21
Between 100,000 and 149,000	3	16
Between 150,000 and 199,000	3	16
Above 200,000	3	16
Unanswered	2	11
Marital status (*n* = 19)		
Married	14	74
Single	2	11
Divorced	1	5
Living common law	2	11
Employment status		
Employed	18	75
Unemployed	5	21
Retired	1	4
Region of origin		
North American	3	13
British Isles	2	8
Northern European	1	4
Southern European	2	8
Western European	3	13
Eastern European	2	8
Other European	1	4
Latin, Central, South American	4	17
Caribbean	2	8
Arab	1	4
French Canadian	2	8
Unanswered	1	4
Number of affected children (*n* = 19)		
1	16	84
2	3	16

Patient Characteristics	Total (*n* = 22)	%

Patient sex		
Female	12	55
Male	10	45
Patient age (years)^†^		
1–4	3	14
5–9	9	41
10–19	6	27
20–34	4	18

Abbreviations:

CAD = Canadian dollar

LD = Leukodystrophy

This table summarizes parental and patient demographic characteristics including parents' sex, preferred language, continent of residence, distance from closest LD center or clinic, household income, marital status, employment status, region of origin, number of children with POLR3-HLD, and the sex and age of the patients.

* Demographic values are represented as a function of total parent participants (*n* = 24) or total qualitative interviews (*n* = 19).

† Patients' ages in years are those at the time of the interview.

**Table 2.** Themes and Representative Quotes

Themes	Quotes*

Existing barriers in accessing care	
Travel distance, coordination, and long wait times	“That you know, that is always so shocking to me, sometimes they don't have appointments for six months. And I think, I don't know what I'm going to be doing in six months. She's healthy now, you should see her now. And I do have to cancel quite a few appointments because the wait is just so long.” (Parent 5)
Public versus private health care systems	“So, I just got a letter in the mail two or three days ago saying, ‘Oh, whoops, we can't cover MTL1281's medication anymore because we don't see a need for it’. Like, what do you mean? I mean it keeps her lungs from collapsing, it kind of seems a little important to me.” (Parent 11)
Positive influences on access to care	“Well, one of the strong points for me is that with telemedicine appointments I don't always have to take a day off, I could organize my schedule so as not to lose a day's pay, and still have access to a service.” (Parent 14A)
Limited knowledge in diagnosis and care	
Limited knowledge and support	“But I remember calling the neurologist kind of yelling, crying, saying that we needed more support. We needed more support. I was the one pushing for that since the very beginning. There was like no information packet set up to help guide us.” (Parent 6)
Perceived downfalls in care	“And then his pediatrician got us started with physiotherapy and the therapist was like, well, it could be, cerebral, or not cerebral palsy, muscular dystrophy, or it could be, […] and she named a few things. Leukodystrophy wasn't even on the radar. I don't even think that she knew what it was.” (Parent 15A)
Paternalism	“It took about 7 to 8 months before his pediatrician actually listened to us. He kept dismissing our concerns, saying that he was going to grow out of it, not to be concerned. But at this point, as you know, he's my third child. I know what a healthy child looks like at the age of 3–4 years old, and this wasn't it.” (Parent 8)
Parents as experts and advocates of their child(ren)'s care	
Parents must be informed and proactive	“And you know, I work part-time. I mean, I'm so lucky that like, Tuesdays and Fridays, I dedicate to advocating for MTL1243, getting through my emails, talking to you, you know? We met with the Social Security or the disability services last Tuesday. You know, people are like, what do you do? I'm like, oh my God, Tuesdays and Fridays are not a day off. That is my advocating, caring for MTL1243, and making sure he's getting to all of his appointments”. (Parent 3)
Insufficient communication in health care	“I'm constantly passing messages on. I've had MTL1211's physiotherapist write me letters to take to an orthopedic appointment and I'm there thinking, why can't you contact each other? Yeah, it's crazy.” (Parent 1)
The benefit of parental background	“I'm appreciative that they listen, but I worry about other families, like with my nursing background, it's like I have an advantage and I know how to advocate and like push some of these things, but I feel like if I didn't have that maybe his care would be more lacking.” (Parent 4)
Perceived superior care by leukodystrophy specialists	
The support found in an expert	“Whereas Dr. […], when she delivered the news to us, she said, you know, MTL0661 is going to live a very long life and I urge you to treat your child like you would your other two neurotypical children. You know, if he gets in trouble, you bring it to his attention, if he does well in school, you treat him like normal. He's going to live a long life, as long as you and your husband are on the same team, and you recognize some of these symptoms. He's going to be with you for a really long time and try to give him the best quality of life that you can. And that is how she delivered that news. You see the difference? She gave me hope. You know what I mean? Whereas the other doctors didn't.” (Parent 8)
The benefit of specialized expertise	“Being in a facility that specializes in the diagnosis that you are seeking services for, I guess spiritually or emotionally feels better because you feel understood and they kind of know what you're going through and you know they deal with it on a daily basis so, […] it's like a different type of support that you feel when you're in an environment that has so much knowledge and experience.” (Parent 9B)

Abbreviation:

POLR3-HLD = POLR3-related hypomyelinating leukodystrophy

This table provides representative quotes from the interviews with parents of patients with POLR3-HLD.

* Some quotes have been translated from French to English and all have been edited for clarity.
