# Supplementary material for: POLR3-Related Leukodystrophy: A Qualitative Study on Parents’ Experiences With the Health Care System
Source: Pediatr Neurol. Author manuscript; Available in PMC 2026 Feb 27. (PMC12289442; doi:10.1016/j.pediatrneurol.2025.02.011)
Supplement: Supplemental Data [file NIHMS2095914-supplement-Supplemental_Data.docx]

**SEMI-STRUCTURED INTERVIEW GUIDE**

*Perceived QoC: Quality of Care*

**Introduction**

Hello,

First, I would like to thank you on behalf of the MyeliNeuroGene Lab for participating in this qualitative interview study. We are completing these interviews because we are trying to get a better understanding of your experience with the healthcare system as a parent of a child with POLR3-related leukodystrophy (4H leukodystrophy). We’re doing this interview with you because you’re in the best position to provide us with insights about what needs to be improved in the future. I will be asking you some questions about the healthcare and services your child has received since they have been ill. I will also ask some questions about your life at home. Audio and visual or audio only will be recorded from this interview. After the interview, I will transcribe our conversation to try to find themes that are common among different families in order to identify the common problems in healthcare services. Some of the questions may be personal or make you feel uncomfortable. Please say “pass” if you wish to skip any questions. Your answers are completely confidential, and when we transcribe this conversation, your name will be removed.

Do you have any questions?

**Preface**

When you are ready, I will start recording.

*[Start recording]*

*[State your name and the name of the parent. State the date of the interview.]*

I would like to begin by getting to know you and your family a little better.

1. To start, can you tell me a little bit about your family?
2. How are you related to [the patient]?
3. Who is the primary caregiver in the family for [the patient]?

**Patient Diagnosis**

I would first like to discuss the diagnostic period. So, this includes the period while seeking a diagnosis and the moment of actually receiving it.

1. Which symptoms did your child first experience that led you to seek a diagnosis?
2. How long did it take between the onset of symptoms and receiving a diagnosis?
3. Was there any diagnostic delay?
   1. If so, how long was the delay?
4. Was the patient initially misdiagnosed?
   1. Please specify the initial misdiagnosis.
5. How did you feel before the diagnosis (after the onset of symptoms) and after the diagnosis?
6. Describe the event of receiving your child’s diagnosis …
   1. Who provided the diagnosis? (Primary physician, neurologist, genetic counsellor, etc.)
   2. Did you feel supported in that moment? (i.e., was any additional explanation provided about the disease and your next steps?)
   3. Who helped you understand this new diagnosis?
7. How would you rate your knowledge level of 4H leukodystrophy prior to your child’s diagnosis compared to now? How about your friends and family?

**Patient Care**

So, we will now move on to discuss some aspects regarding the healthcare that your child(ren) currently receive(s).

1. What neurological and non-neurological complications does your child experience?
2. What is the name of the hospital your child is primarily followed at?
   1. Is this at a leukodystrophy center or clinic?
   2. What services do you receive at this hospital?
   3. How far do you travel to be seen there?
   4. How often are you seen there and how long do you spend there?
3. Are you aware of the closest leukodystrophy center/clinic to you?
   1. Have you ever been assessed there or thought of bringing your child there? Why or why not?
   2. How would you describe your experience at a leukodystrophy center/clinic, and with the physicians there? Did you find it to be beneficial?
4. What does your child’s team of specialists look like?
   1. How far must you travel to see each of them?
   2. How often do you see each specialist?
   3. Were you referred to these specialists or did you have to find them on your own?
      1. How long did it take to be referred?
      2. How long did it take to be seen by the specialists?
   4. Did you begin seeing them before or after the official diagnosis?

**For parents of older children:**

1. Describe any change that has occurred as your child has aged:
   1. Regarding your child’s condition.
   2. Regarding the healthcare they receive.
   3. Regarding the personal care you provide to them.

**Access to Care**

Now, I would like to discuss your experience accessing said healthcare.

1. What has your experience accessing healthcare for your child been like? How has this differed from accessing specialized leukodystrophy care?
2. How comfortable are you in navigating the healthcare system now? How does this differ from the start?
3. Did you face any barriers when seeking specialized care for your child?
   1. How much time do you spend searching for and contacting physicians?
   2. How much time do you spend travelling to and attending appointments?
   3. Does your financial situation influence how and what care you access?
4. How are you managing your child’s daily care and life’s other activities?
5. What have you learned from this experience?
6. Do you have anyone helping you in this? Who is your biggest ally?

**Perceived Quality of Care**

These next questions will focus on the perceived quality of care that your child(ren) receive(s) (i.e., your opinion on the quality of care).

1. How would you describe the care that your child receives?
   1. By their primary physician?
   2. By their specialists?
2. How would you describe the care your child receives at a leukodystrophy center/clinic?
3. How would you rate physician knowledge of your child’s diagnosis and disease? How does this compare to those at the leukodystrophy center/clinic?
4. How would you rate your understanding of your child’s condition compared to their physicians?
5. Who do you see as your primary point of contact for your child’s care?
   1. Do they oversee your child’s care and track the progression of their condition?
   2. How would you rate the communication between yourself and them?
6. How do you feel about the communication between specialists and coordination of treatment plans?
7. Do you feel the need to be an expert and advocate for your child’s healthcare needs?
   1. Do you believe this effort has made an impact on the care received by your child?

**Thoughts on Healthcare**

I would like to discuss some of your general thoughts and perceptions of healthcare.

1. Could you describe your perception of healthcare?
2. Are you satisfied with the healthcare your child receives?
3. How do you think healthcare for your child could be improved?
4. What aspect of your child’s healthcare do you think most important?

**Ethnicity and Geographical Location**

I would like to briefly discuss what your opinion and experience has been accessing healthcare for your child(ren), and how your ethnicity and geographic location has impacted that.

1. Do you feel that your ethnicity and/or geographical location and that of your child has affected how you navigate healthcare, your access to care or the QoC your child receives?

**Telemedicine**

During the COVID-19 pandemic, we had seen a great shift in the implementation of telemedicine and virtual healthcare appointments, similar to how we are discussing over Zoom, Teams, or WhatsApp today, and some healthcare providers continue to do so.

1. Have you used telemedicine in the past? How frequently?
   1. Was this with a leukodystrophy specialist or primary physician?
2. From your experience is the quality of care received online superior, inferior or the same as in-person visits?
3. What is the biggest highlight and lowlight of using telemedicine?
   1. Any suggested improvements?
4. Has the recently increased use of telemedicine affected your access to care?

**Parental Needs**

Before we wrap up, I just have one last question for you.

1. As a parent of a child with POLR3-HLD, do you have any unmet needs that you think could be provided by the healthcare system or your personal support networks?

**Closing Remarks**

1. Is there anything else you would like us to know?
